# Gene × gene and gene × environment interactions for complex disorders

**DOI:** 10.1186/1753-6561-1-s1-s72

**Published:** 2007-12-18

**Authors:** Robert Culverhouse, Anthony L Hinrichs, Carol H Jin, Brian K Suarez

**Affiliations:** 1 Department of Medicine, Washington University, 660 South Euclid, GMS-Box 8005, St. Louis, Missouri 63110, USA; 2 Department of Psychiatry, Washington University, 660 South Euclid, Box 8134, St. Louis, Missouri 63110, USA; 3 Department of Genetics, Washington University, 660 South Euclid, Box 8134, St. Louis, Missouri 63110, USA

## Abstract

The restricted partition method (RPM) provides a way to detect qualitative factors (e.g. genotypes, environmental exposures) associated with variation in quantitative or binary phenotypes, even if the contribution is predominantly an interaction displaying little or no signal in univariate analyses. The RPM provides a model (possibly non-linear) of the relationship between the predictor covariates and the phenotype as well as measures of statistical and clinical significance for the model.

Blind to the generating model, we used the RPM to screen a data set consisting 1500 unrelated cases and 2000 unrelated controls from Replicate 1 of the Genetic Analysis Workshop 15 Problem 3 data for genetic and environmental factors contributing to rheumatoid arthritis (RA) risk. Both univariate and pair-wise analyses were performed using sex, smoking, parental DRB1 HLA microsatellite alleles, and 9187 single-nucleotide polymorphisms genotypes from across the genome. With this approach we correctly identified three genetic loci contributing directly to RA risk, and one quantitative trait locus for the endophenotype IgM level. We did not mistakenly identify any factors not in the generating model. All the factors we found were detectable with univariate RPM analyses. We failed to identify two genetic loci modifying the risk of RA. After breaking the blind, we examined the true modeling factors in the first 50 data replicates and found that we would not have identified the additional factors as important even had we combined all the data from the first 50 replicates in a single data set.

## Background

Many diseases important to public health are not due solely to a single mutation or environmental insult. Instead, complex interactions among multiple genes and environmental exposures likely play crucial roles in the etiology of diverse phenotypes from schizophrenia to chemotherapy response.

The restricted partition method (RPM) [1] is an algorithm for identifying qualitative genetic and environmental factors contributing to quantitative or binary phenotypes, even if the effects are largely due to an interaction displaying little or no signal detectable by univariate analyses [2,3]. The RPM is a multivariable extension of the "measured genotype" [4] approach. If variation at a locus (or combination of loci) contributes to trait variation, different multilocus genotypes can be expected to display different trait means (or for binary data, different proportions of "cases"). In contrast, genotype classes defined by loci that are not in linkage disequilibrium (LD) with loci that contribute directly to trait variation would not be expected to display different mean trait values.

We applied the RPM to the Replicate 1 of the Genetic Analysis Workshop 15 data, blind to the generating model, in order to test the utility of the method for genome-wide association data for a complex trait.

## Methods

The RPM merges multivariate genotype and environmental strata with similar mean trait values (proportions of cases) until all of the remaining groups have significantly different means or all the genotypes are merged into a single group (indicating that the particular loci are not jointly correlated to variation in the trait). If distinct subgroups are found, the proportion of variation attributable to the partition, *R*^2^, is calculated and statistical significance is evaluated using empirical null distributions generated by permutations of the data. Details of the RPM can be found in Culverhouse et al. [1]. Originally designed for quantitative trait data, the RPM has recently been shown to have good power and the nominal rate of false positives when used with binary data [5].

We initially restricted our attention to data from a single replicate (Rep 1). We formed a group of unrelated cases (*N *= 1500) by selecting the most severely affected sib. If both sibs were equally affected, we selected the first sib. This case sample was compared to the *N *= 2000 unrelated controls supplied by the data providers. Potential predictors of phenotype consisted of genotypes from the sparse SNP map (*N *= 9187) along with the covariates sex, smoking, and the DRB1 HLA (henceforth "DR") allele inherited from each parent. This resulted in a total of 9191 predictor variables.

After these analyses, we broke the blind and performed secondary analyses on the first 50 replicates to evaluate the effect of a larger sample or different replicates.

## Results

### Univariate analyses

Univariate RPM analyses, like multivariate RPM analyses, evaluate the different genotype cells for differences in means. Retaining only those factors that resulted in an RPM model with *R*^2 ^> 0.005 reduced the 9191 predictors to a set of 19 SNPs, plus the DR alleles, sex, and smoking. The permutation *p*-values for all these factors were < 0.05 after correcting for 10,000 tests. Of the 19 SNPs identified, the only ones not on chromosome 6 were chromosome 11 SNP 389 and chromosome 18 SNP 269. The list of factors and their corresponding *R*^2 ^are listed in Table 1.

**Table 1:** Univariate RPM results (list of all factors with *R*^2 ^> 0.005 and *p *< 0.05)

Chromosome	Factor	*R*^2^	cM (from SNP 128)
6	SNP 128	0.0104	--^a^
6	SNP 129	0.0138	0.0217
6	SNP 130	0.0138	0.0220
6	SNP 133	0.0053	0.5729
6	SNP 134	0.0153	0.6029
6	SNP 138	0.0170	1.1369
6	SNP 139	0.0170	1.1376
6	SNP 144	0.0052	1.2224
6	SNP 145	0.0084	1.2403
6	SNP 147	0.0070	1.4361
6	SNP 150	0.0062	1.7296
6	SNP 152	0.2098	2.4694
6^b^	SNP 153	0.5163	2.4999
6	SNP 154	0.4580	2.5055
6	SNP 155	0.1073	2.6610
6	SNP 160	0.0094	6.3276
6^b^	SNP 162	0.0330	7.6641
			
11^b^	SNP 389	0.0295	--
			
18^b^	SNP 269	0.0070	--
--			
--	Sex	0.0537	--
--	Smoking	0.0258	--
--	HLA-DR (father)	0.3718	--
--	HLA-DR (mother)	0.3654	--

^a^--, Not applicable.

^b ^Markers identified as independent contributors to RA risk. The other SNPs are in LD with one of the footnoted markers but have lower *R*^2^.

For the SNPs on chromosome 6, the sex-averaged genetic distances from SNP 128 are listed to give an idea of the recombination rates between the markers. We compared these results to what would have been obtained by a traditional χ^2 ^analysis of the data. Except for one SNP, this is exactly the list of factors that had a Bonferroni corrected χ^2 ^*p*-value < 0.05 for genotype differences between cases and controls. The exception is SNP 137 on chromosome 6, which was barely past the threshold in the χ^2 ^analysis. At first there appear to be three *R*^2 ^peaks in this region (SNP 138, 153, and 162). An analysis of LD by Suarez et al. [6] revealed that SNP 138 was in strong LD with SNP 153, but that SNP 162 was in equilibrium with SNP 153. This led us to conclude that there are two distinct factors contributing to RA risk, located near SNP 153 and 162.

### Two-way analyses

Interactions in multivariate RPM analyses are indicated by increased *R*^2 ^values over the univariate analyses, decreased *p*-values (after correction for the increased number of tests), and by the structure of the resultant multivariate model. We do not currently have a formal test for interactions. In part, this is because the main goal of the RPM is to detect factors contributing to phenotype, even if their effects are primarily seen in interactions, rather than to estimate the variance components accurately.

We began our analyses by searching for increases in *R*^2^. Because the *R*^2 ^in these analyses are estimated from a highly ascertained data set, they likely overestimate the proportion of variation explained by these factors in the whole population.

**Figure 1:**
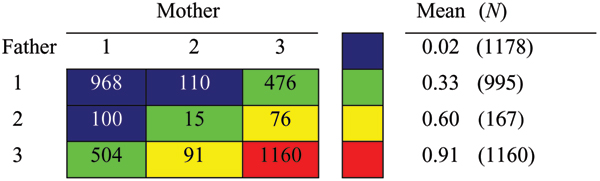
**RPM model for the DR alleles inherited from bothparents (*R*^2 ^= 0.56)**. Mean = proportion of affected in each genotype group;*N *= total number of subjects in each genotype group.

Combining the DR alleles inherited from the mother and the father results in the two-factor model with the highest *R*^2^. Individually, these factors each account for approximately 37% of the trait variation (see Table 1). When analyzed jointly, this jumps to 56%. The model chosen by the RPM is illustrated in Figure 1. The values in the grid indicate the number of subjects in each of the categories, "Mean" gives the proportion of subjects in each group who are affected, and "N" gives the total number of individuals in each group in the final model. The model is symmetric about the main diagonal, indicating that the effect of inheriting a risk allele from either parent is approximately the same. The diagonally banded pattern indicates that the effects of these two factors are approximately additive. This suggests that a single-locus genotypic analysis would provide approximately the same information as keeping the parental alleles as two separate factors. For some later analyses, we followed this approach.

**Figure 2:**
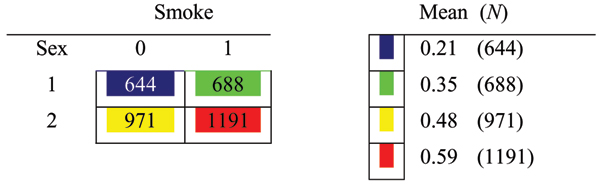
**Model for smoking vs sex. Models approximatelyadditive (*R*^2 ^= 0.078)**. Mean = proportion of affected in each genotype group; *N *= total number of subjects in each genotype group.

A similar additive effect is found between sex and smoking (Figure 2). In this case, all four of the cells are found to be distinctly different from the others by the RPM. By themselves, sex and smoking account for approximately 5.3% and 2.6% of the trait variation, respectively. Jointly, they account for 7.8%.

In particular, we note that the modest univariate signals from chromosome 6 SNP 162, chromosome 11 SNP 389, and chromosome 18 SNP 269 appear to display roughly additive effects, suggesting that they are independent contributors to the phenotype (see Table 2). However, no covariate pair (of the 42,242,645 pairs examined) stood out as providing a large increase in explained variance over the sum of the individual variances.

**Table 2:** Univariate and two-locus models from three secondary peaks

Model	Chromosome	SNP1	Chromosome	SNP2	*R*^2^
Univariate					
	6	162	--^a^	--	0.0330
	11	389	--	--	0.0295
	18	269	--	--	0.0070
					
Two-way					
	6	162	11	389	0.0624
	6	162	18	269	0.0404
	11	389	18	269	0.0354

^a^--, Not applicable.

### Three-way analyses

Although none of our two-way analyses showed a large interaction effect, we identified 88 SNPs that were either individually significant or appeared to slightly increase *R*^2 ^in combination with another predictor. Using this limited set of SNPs, along with the covariates sex, smoking, and DR alleles, we looked for three-way interactions.

The addition of chromosome 6 SNP 153 to the model for the parental DR alleles did not substantially increase *R*^2 ^(0.5601 vs. 0.5611). We evaluated the correlation between haplotypes at these two loci (since haplotypes were available). We found that in cases, allele 1 at SNP 153 was always on the same haplotype as allele 2 or 3 at the DR locus, while allele 2 at SNP 153 was on the same haplotype as allele 1 from the DR locus 98.9% of the time (χ^2 ^= 2962.3). Only four of the six possible haplotypes were observed, and one was extremely rare. In controls, the correlation is similarly high (χ^2 ^= 3990.6). This suggests that chromosome 6 SNP 153 is merely a surrogate for DR.

In contrast, other factors in combination with DR could produce a modest increase in *R*^2^. The RPM gave evidence of sex and chromosome 11 SNP 389 being contributing factors independent of the DR locus. Both increased the explained variation from 56% to 58%. The model including sex and DR resulted in slightly different groups for the two sexes, suggesting a possible small, non-additive interaction.

After we performed these analyses, we combined the two DR alleles into a single DR genotype, thereby reducing analyses involving DR by one dimension. Using DR genotypes produced almost identical results as treating the alleles as separate factors, so we will only present results from using DR genotypes in Table 3. This table lists the one-, two-, three-, and four-factor models providing the highest explained proportion of trait variance.

**Table 3:** Best^a ^one-, two-, three-, and four-factor models

Model	*R*^2^
DR	0.5601
DR+sex	0.5801
DR+Chr 11-SNP 389	0.5801
DR+sex+Chr 11-SNP 389	0.6006
DR+sex+Chr 11-SNP 389+Chr 18-SNP 269	0.6154

^a^Models explaining the highest proportion of variance.

### After the model was known

Once the model was known, we discovered that the factors we identified from our univariate analyses (see Table 1) were all part of the RA risk model (although the chromosome 11 SNP was indirectly related by being a quantitative trait locus (QTL) for the endophenotype IgM). We found that we had missed two genetic loci that affected RA risk, one on chromosome 16 and one on chromosome 8. We combined the data from 50 replicates (for a total of 75,000 cases and 100,000 controls) to determine if an increase in sample size would allow these two factors to be detected. We found that this was not the case. The locus on chromosome 8 was at the end of the chromosome, 3.5 cM past the last marker, and appears not to be in LD with the nearest markers. In contrast, the locus on chromosome 16 did have a marker within 0.01 cM, but (perhaps due to the strong ascertainment) the effect was too subtle to detect even with such a large data set.

## Discussion

Two related and appealing properties of the RPM are that it provides a measure of effect size (*R*^2^) in addition to a measure of statistical significance (*p*-values) and that it proposes a model of how the predictive factors affect the phenotype.

Effect size is related to clinical importance and should be considered alongside evidence of statistical significance whenever medical researchers need to decide which signals should be pursued in further studies. We used *R*^2 ^in two ways: i) as a threshold to filter out factors of little clinical interest and ii) as a method to localize a signal. In practice, the decision of how small an effect of clinical interest is depends on the phenotype, the researcher, and funding, and should be made in consultation with a skilled clinician. In this case, we picked a low threshold, *R*^2 ^≥ 0.005. Because this only identified a small number of markers of interest, we stayed with that threshold. As a result, we were able to identify a QTL related to our phenotype of interest without picking up any false-positive signals. In this we were lucky. Our second use of *R*^2 ^was to localize a signal within regions of LD on chromosome 6. Because of the LD, several markers (see Table 1) had permutation-based *p*-values < 0.05 after Bonferroni correction. Because of the computational expense of permutation testing, instead of increasing the number of permutations by multiple orders of magnitude to distinguish between them, we used *R*^2 ^and LD patterns to identify the two SNPs most correlated to the causative loci on chromosome 6.

The value of the modeling of the phenotype was shown in our examination of the DR alleles. Although these were presented in the data set as separate maternally and paternally inherited alleles, the RPM model suggested that parental origin did not make much difference. As a result, we were able to reduce the dimensionality of some analyses that included DR as a factor.

Drawbacks of using the method to test genome-wide association data for main and interaction effects include the computational burden of permutation testing, the ad hoc choice of what should be considered a significant increase in explanatory variance when an additional factor is added to the model, and the problem of multiple testing correction.

## Conclusion

Using the RPM for univariate analyses on a single data replicate, and blind to the generating model, we were able to identify four genetic factors that directly contributed to RA status and one genetic locus that was a QTL for an associated endophenotype, IgM. We did not detect any false-positive signals.

The *R*^2 ^values provided by the algorithm were useful in localizing causative loci within regions of LD on chromosome 6 and in providing a filtering mechanism to eliminate small effect false positives.

Multilocus analyses were useful in determining that DR, chromosome 6-SNP 162, chromosome 11-SNP 389, and chromosome 18-SNP 269 were independent contributors to RA risk, and that the DR alleles displayed a dose effect, with no parent-of-origin effect. Because of the extremely high correlation between DR and chromosome 6-SNP 153 alleles, we failed to deduce that they were associated to two distinct contributing loci.

Two genetic factors contributing to RA risk were not detected. One, on the end of chromosome 8, was apparently not in LD with any of the marker SNPs. Even with knowledge of the generating model, we did not find evidence of either of these factors in an ascertained sample of 75,000 cases and 100,000 controls taken from 50 replicate data sets.

## Competing interests

The author(s) declare that they have no competing interests.
